# An Update on Multimodal Ophthalmological Imaging of Diffuse Choroidal Hemangioma in Sturge–Weber Syndrome

**DOI:** 10.3390/vision7040064

**Published:** 2023-10-06

**Authors:** Chiara Ciancimino, Mariachiara Di Pippo, Daria Rullo, Francesco Ruggeri, Flaminia Grassi, Gianluca Scuderi, Solmaz Abdolrahimzadeh

**Affiliations:** Ophthalmology Unit, Neurosciences, Mental Health, and Sense Organs (@NESMOS) Department, Faculty of Medicine and Psychology, University of Rome Sapienza, St. Andrea Hospital, 00189 Rome, Italy; chiara.ciancimino@uniroma1.it (C.C.); mariachiara.dipippo@uniroma1.it (M.D.P.); daria.rullo@uniroma1.it (D.R.); fra.ruggeri@uniroma1.it (F.R.); flaminia.grassi@uniroma1.it (F.G.); solmaz.abdolrahimzadeh@uniroma1.it (S.A.)

**Keywords:** diffuse choroidal hemangioma, Sturge–Weber syndrome, multimodal imaging, fundus, enhanced depth imaging, spectral domain optical coherence tomography (OCT), near-infrared reflectance (NIR)

## Abstract

Sturge–Weber syndrome (SWS) is characterized by facial port-wine stains, leptomeningeal hemangiomas, and prominent ocular manifestations such as glaucoma and diffuse choroidal hemangiomas (DCHs). Imaging modalities are critical for diagnosing and longitudinally monitoring DCHs in SWS. Fundus photography is fundamental in assessing both eyes simultaneously, fluorescein angiography and indocyanine green angiography effectively map the retinal and choroidal circulation, and ultrasonography offers essential structural insights into the choroid and retina. NIR imaging reveals subtle retinal pigment changes, often overlooked in standard fundus examination. Enhanced depth imaging spectral domain optical coherence tomography (EDI-SDOCT) and swept-source OCT (SSOCT) improve the visualization of the choroidal-scleral boundary, essential for DCH characterization. The potential of OCT angiography (OCTA) is under exploration, particularly its role in predicting signs of disease progression or worsening, as well as potential new biomarkers such as the choroidal vascularity index (CVI). The present review aims to provide an update on multimodal imaging of DCHs in SWS.

## 1. Introduction

Sturge–Weber syndrome (SWS) is a rare neurocutaneous disorder characterized by facial angiomatosis, also known as nevus flammeus or port-wine stain (PWS), leptomeningeal hemangiomas, and ocular manifestations including glaucoma and diffuse choroidal hemangiomas (DCHs). This triad of symptoms was first described by William Allen Sturge and Frederick Parkes Weber in the late 19^th^ century [1,2]. After almost a century, Roach proposed a classification with three distinct phenotypes for SWS patients: Type 1, which is the classical form including facial angioma, leptomeningeal angiomas, and possible glaucoma; type 2, with facial angioma and possible glaucoma; and type 3 also known as forme fruste presenting leptomeningeal angiomas in the absence of facial angioma [3,4,5].

The incidence of SWS is approximately 1 in 20,000 to 50,000 live births, affecting both males and females equally [6]. The syndrome is commonly sporadic and nonfamilial, with no identified hereditary pattern or predisposition [1,2]. In 2013, Shirley et al. initially pinpointed somatic mosaic alterations in the GNAQ gene as the underlying cause of both PWS and SWS. These genetic changes disrupt the RAS effector pathway, prompting an increase in cellular growth and hindering programmed cell death. The onset of this mutation is likely more premature in SWS compared to isolated PWS leading to a broader spectrum of clinical manifestations [7].

PWS is a more common congenital vascular malformation with an incidence of approximately 1 in 300. Infants with PWS have a chance of approximately 6% of having SWS [8,9]. Previously, the trigeminal distribution of the PWS was identified as a risk factor for the association with SWS. However, more recent dermatological studies conducted by Waelchli et al. demonstrated how PWS in the forehead region was associated with a greater risk of associations with SWS [9].

Diverse neurological manifestations have been described in patients with SWS. Most frequently, patients are affected by focal seizures that are often contralateral to the facial PWS. Patients may also experience hemiparesis and cognitive impairment with varying severity [9].

Various elements contribute to cognitive deterioration in SWS patients: A history of seizures spanning over two years, the presence of leptomeningeal angiomas on both sides, the onset of symptoms before the age of one, calcifications in the brain, and angiomas on the left leptomeningeal region. It is imperative to manage and control seizures early on to prevent cognitive setbacks. [10,11]. SWS patients rarely have intracranial hemorrhages, and there is little evidence of acute cerebral ischemia [12].

**Figure 1:**
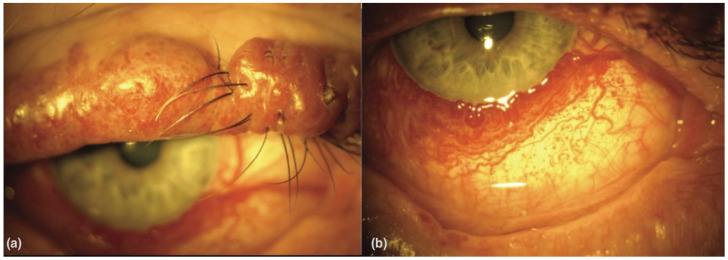
(**a**) Port-wine stain of the upper lid with nodularity in a patient with Sturge–Weber syndrome. (**b**) Diffuse conjunctival vascularity in a patient with Sturge–Weber syndrome. From [15].

Ocular manifestations are present in approximately 50% of SWS patients. Glaucoma and DCHs are most common; however, diverse ocular manifestations affecting different eye structures have been documented. These include PWS affecting the eyelids as shown in Figure 1a, conjunctival telangiectasia as in Figure 1b, iris discoloration with heterochromia or vascularization, retinal arteriovenous aneurysm, and optic nerve abnormalities. Patients may also experience visual disturbances such as strabismus, nystagmus, and cortical blindness [13,14].

When only the upper eyelid is involved, glaucoma has a frequency of 21%, but this rises significantly to 72% when the facial PWS involves both eyelids [9,16,17,18]. Moreover, in SWS, the eye on the same side as the PWS typically exhibits glaucoma [19]. The origins of glaucoma in SWS are complex, with two primary theories. The first theory suggests a mechanical origin, attributing it to deformities in the anterior chamber angle, which hinder the aqueous humor’s natural outflow. The second theory postulates that elevated episcleral venous pressure is linked to arteriovenous shunts in patients with episcleral hemangioma [20,21,22,23].

Rarely, angle-closure glaucoma in SWS is associated with specific ocular manifestations, including edema of the ciliary body, ciliochoroidal effusion, anterior rotation of the ciliary body, and inflammation of the lens leading to acute glaucoma due to the forward movement of the iridolenticular diaphragm [24]. Additionally, ciliochoroidal effusion has been linked to various disorders, such as venous congestion, ocular inflammation, neoplastic and systemic diseases, and traumatic events [25,26].

Managing glaucoma in SWS involves a combination of medical and surgical approaches. Latanoprost has been found to effectively control intraocular pressure (IOP) in approximately 50% of SWS glaucoma cases [23]. Additionally, topical beta-blockers and carbonic anhydrase inhibitors have shown benefits in cases with reduced outflow and without buphthalmos [23]. When medical therapy is insufficient in controlling IOP, surgical interventions become necessary. Trabeculotomy and goniotomy, as well as trabeculectomy, posterior lip sclerectomy, and trabeculotomy-trabeculectomy, are among the reported surgical treatment modalities for SWS-related glaucoma [23,27]. Moreover, using Ahmed valve implants, Molteno tubes, and CyPass suprachoroidal shunts has been considered to provide better long-term outflow and minimize the risk of complications associated with traditional trabeculectomy [23,28,29,30,31].

DCHs affect around 19–71% of SWS patients; this manifestation is typically unilateral and ipsilateral to the PWS [32]. DCHs arise from abnormal collections of blood vessels within the choroid, leading to vascular congestion and potential complications such as retinal detachment and subretinal fluid accumulation.

Multimodal imaging methods are vital in facilitating the diagnosis and management of DCHs in SWS. Various imaging modalities have been employed, including fundus photography, fundus autofluorescence (FA), fluorescein angiography (FFA), indocyanine green angiography (ICGA), spectral domain optical coherence tomography (SDOCT), and ultrasound imaging [7,31,32,33,34,35]. These imaging techniques provide valuable information about the morphology, vascularity, and associated changes in choroidal hemangiomas, enabling timely interventions and appropriate management strategies to preserve visual function and mitigate potential complications.

In this narrative review, we aim to provide a comprehensive overview of the role of multimodal imaging methods in the diagnosis and management of DCHs in SWS. We discuss the clinical features and imaging findings for DCH. Integrating various imaging techniques with clinical evaluation is crucial for accurate diagnosis and optimal management of patients with SWS [7,31,32,34,35].

## 2. Discussion

### 2.1. Histopathological Findings and Clinical Characteristics of Choroidal Hemangiomas in Sturge-Weber Disease

Choroidal hemangiomas are uncommon vascular malformations that tend to increase in size due to internal venous obstructions rather than cellular proliferation [36,37,38]. These hemangiomas are differentiated by their vascular composition into cavernous, capillary, or mixed [38,39]. The cavernous variety is characterized by expansive vessels with a flat endothelial surface and minimal connective tissue. Conversely, the capillary variety contains smaller vessels set apart by a more dispersed connective matrix. The mixed form is a combination of both types and is the most prevalent in DCHs [38,39].

Circumscribed choroidal hemangiomas (CCHs) are isolated unilateral tumors without systemic association [38], whereas DCHs typically involve more than one quadrant of the choroid and represent the pattern associated with SWS [38,39]. Clinically, a DCH carries significant implications due to its potential impact on visual function and ocular health. Retinal alterations such as micro drusen have been reported, and with tumor enlargement, severe complications such as macular edema and exudative retinal detachment leading to vision loss may occur [39,40,41].

### 2.2. Fundus Photography

Clinical examination with fundus ophthalmoscopy is not always sufficient to diagnose a DCH because patient collaboration is often scarce due to young age and/or cognitive impairment. The comparison of fundus color between the affected and fellow eye is usually indicative when photographic images are compared simultaneously, and shallow DCHs do not significantly alter the fundus appearance [15,42].

**Figure 2:**
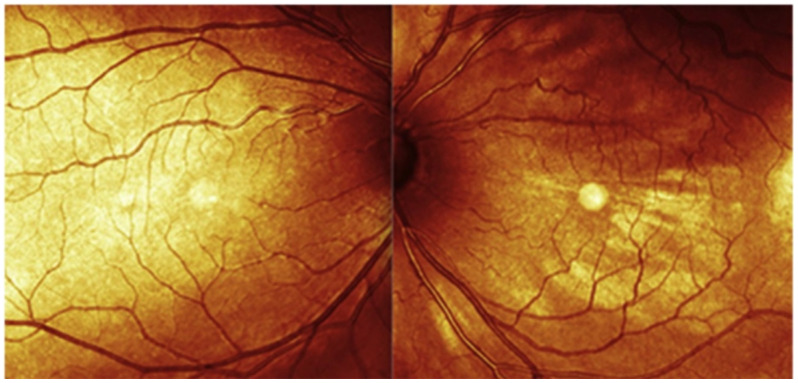
Color photography of the fundus oculi. There is only a mild difference in color between the two eyes. In the left eye (LE), there is evident venous congestion of the left upper venous branch. From [6].

Compared to traditional fundus examination, fundus photography provides several advantages. One of the key benefits is the ease of quickly capturing high-quality images of the fundus compared to performing a fundus examination, which can be complicated, particularly in young children. Fundus photography overcomes the limitations of a conventional fundus examination, where only one eye can be observed at a time. With fundus photography, both eyes can be assessed simultaneously, allowing for a direct comparison of findings between the affected and unaffected eyes. This is particularly valuable in the case of DCH, as the subtle color differences known as “tomato ketchup” appearance and other possible characteristics such as exudative retinal detachments, venous congestion, or hyperplastic retinal pigment epithelium (RPE) changes can be more easily appreciated and documented for longitudinal assessment [42,43]. Figure 2 shows how mild the difference can be with subtle color changes between the affected and fellow eye. These changes would be hard to notice with fundus ophthalmoscopy.

In the context of SWS, where early detection of DCHs and timely intervention are crucial, fundus photography offers an efficient method for assessing and monitoring these hemangiomas longitudinally.

### 2.3. Fundus Autofluorescence (FA)

FA imaging captures light emission from lipofuscin within the RPE [44]. The use of FA imaging has gained prominence as a non-invasive tool to visualize retinal disorders, particularly those marked by elevated autofluorescence due to heightened fluorophores within the RPE.

Untreated DCHs exhibit intrinsic iso-FA or hypo-FA patterns. Conversely, treated choroidal hemangiomas often display a higher prevalence of hypo-FA traits. Furthermore, extrinsic DCH findings, including RPE hyperplasia, atrophy, and fibrous metaplasia, are often associated with hypo-FA patterns. The presence of hyper-FA due to overlying orange pigment (lipofuscin) has also been documented [44,45]. FA imaging provides a non-invasive window to understand the intricate interactions of autofluorescent molecules within the RPE that are of value in evaluating secondary characteristics related to DCH, although it does not give clear indications of the extent or thickness of the hemangioma.

### 2.4. Fluorescein Angiography (FFA)

Fluorescein dye is administered intravenously and allows for a series of sequential photographs that produce an angiographic representation of the retinal vascular structure. This procedure aids in visualizing retinal blood flow patterns, assessing inner blood-retinal barrier integrity, and examining details of the RPE [46]. While FFA is pivotal for diagnostic and research purposes, opinions on its utility in DCHs are controversial. Some authors emphasize that FFA, by itself, may not be entirely diagnostic of DCH.

Within the very first seconds of the examination, a choroidal blush with strong fluorescence is visible and then diminishes rapidly. This pattern of initial fluorescence in DCHs is similar to that observed in CCHs, albeit with broader and more variable involvement. During FFA, the RPE has a screening effect on the choroidal structure preventing a proper analysis of this tissue. Furthermore, there can be retinal detachments or subretinal fluid, which further cover the choroid. Consequently, FFA alone might not consistently identify DCHs from other conditions [45,47,48,49].

However, contrasting opinions argue that FFA can be particularly informative in cases where a DCH is associated with retinal detachment. For instance, FFA has been shown to depict early hyperfluorescence and subsequent late-phase leakage consistent with exudative retinal detachment linked to DCH. This can be especially helpful in conditions with small exudative detachments and therefore help in earlier diagnosis. In another context, FFA uncovers widespread staining with slight leakage across the nodular sections of choroidal tumors, assisting in the detailed depiction of DCHs [50,51].

In conclusion, FFA remains a pivotal tool for investigating retinal vascular dynamics while opinions diverge on its standalone diagnostic efficacy for DCH. There is consensus that the role of FFA is more pronounced when a DCH is associated with retinal detachment. Collaborative utilization with complementary diagnostic methods can optimize the accurate diagnosis and management of DCH-related conditions.

### 2.5. Indocyanine Green Angiography (ICGA)

ICGA employs indocyanine green, which is a dye with a high affinity to plasma proteins, predominantly albumin. This binding enhances the solubility of the dye in blood and allows it to circulate through the vascular system, offering a clear view of choroidal circulation dynamics when exposed to near-infrared light during imaging. In contrast, FFA uses fluorescein dye, which remains within the retinal vasculature and is visualized using blue light. ICGA’s longer wavelength and ability to penetrate deeper layers make it particularly suitable for studying choroidal circulation [52].

ICGA offers a detailed dynamic visualization of DCH. Within the initial imaging phase (10–15 s), the vascular network within the hemangioma is quickly filled, generating a delicate lace-like pattern, which overlays the usual choroidal pattern and diffusely spreads throughout the posterior pole. From around 20–180 s, complete dye filling yields intense hyperfluorescence with a mix of hyper- and hypofluorescent dots on an already luminescent background. Concurrently, areas of diminished choroidal circulation in the upper or lower mid periphery emerge, likely attributed to the tumor’s space-occupying effect, which masks the standard choroidal ICGA pattern [48,49,52].

In the latter phases of ICGA, a pronounced hypofluorescence of the tumor when compared to the adjacent choroid emerges as a signature feature, potentially denoting a diagnostic marker for choroidal hemangioma. This “wash-out phenomenon”, described by Shields et al., may arise from the faster bloodstream in the tumor, leading to more rapid ICGA clearance than its surrounding tissues [31,49].

In summary, ICGA provides valuable insight into the intricate choroidal circulation within DCHs [48,49,52]. However, it is also important to underline that FFA and ICGA are imaging techniques requiring dye administration. These methods are invasive and rarely can give rise to allergic reactions and interfere with renal and hepatic metabolisms, aspects that need to be considered when choosing the type of imaging protocol necessary in studying DCHs.

### 2.6. Spectral Domain Optical Coherence Tomography (SDOCT)

Optical coherence tomography is a non-invasive approach that has revolutionized our ability to study the retina, choroid, and optic nerve with exceptional detail, facilitating a comprehensive retinal and choroidal thickness analysis [53,54,55]. It aids in identifying significant aspects including subretinal fluid, small-sized detachments of the retina, or macular edema, all of which hold critical diagnostic and treatment planning implications for DCH.

**Figure 3:**
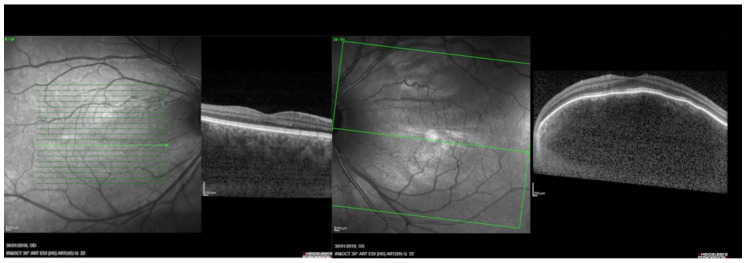
Enhanced depth imaging (EDI) spectral domain optical coherence tomography (SD-OCT) shows bilateral diffuse choroidal thickening, more marked in the left eye (LE) with a dome-shaped macular profile. There are no structural alterations of the retinal layers. From [6].

More advanced SDOCT technology has emerged, particularly enhanced depth imaging (EDI). EDI-SDOCT uses the same light source as standard SDOCT, with a wavelength range of 840–870 nm. EDI-SDOCT is a technique in which the device is closer to the eye, allowing the inverted image to visualize the choroid in better detail. Thus, the enhancement of depth imaging is not because of a change in wavelength, but rather due to the approach [56,57,58,59]. This approach enables the analysis of the structure of the choroid with an impressive resolution of 3–4 microns, allowing for better measurements of choroidal thickness (CT), defined as the region between Bruch’s membrane and the external choroid-scleral boundary. Notably, normal CT can vary significantly; in an age range of 10–30 years old, CT ranges from 167.9 to 310 microns [59]. This parameter is affected by age, gender, axial length, refractive error, and systemic health conditions [60,61,62]. In Figure 3, an interesting case of bilateral DCHs in a patient with SWS is shown; here the clear identification of the choroidal-scleral boundary is not possible for the right eye, while it seems more visible on the left eye, thus it is possible to appreciate an increased CT in both eyes even if not readily measurable as in Figure 3.

Choroidal vasculature is visualized on EDI-SDOCT as hyperreflective spots underneath Bruch’s membrane [45]. CT measurements in SWS were reported firstly by Arora et al. in 2013, where thicker choroids were measured in eyes with a DCH compared to the fellow healthy eye [63]. This parameter was further analyzed by Abdolrahimzadeh et al. in 2016, where an increase in mean CT was measured both in the affected (561.6 μm) and fellow eye (322.0 μm) when compared to healthy age-matched control subjects [62]. A similar study on CCHs showed similar results with increased subfoveal CT in the affected and fellow eye compared to age-matched healthy subjects [64]. The relevance of this parameter measured with EDI-SDOCT is also in following patients after treatment, such as in a study performed by Cacciamani et al., where CT was reduced in eyes affected by a DCH following PDT therapy [56]. Aside from its ability to assess retinal and choroidal thickness, SDOCT plays a pivotal role in identifying additional characteristics of DCH. It can reveal the breakdown of Bruch’s membrane and RPE, often linked to lipofuscin deposition, intraretinal fluid, and subretinal fluid. Additionally, SDOCT detects fibrous transformation in over half of DCH cases, particularly in the proliferated RPE. It also highlights thinning of the RPE-photoreceptor layer (RPE-PHL) and changes resembling pachychoroid pigment epitheliopathy. These insights aid in comprehensive diagnosis and personalized treatment planning [42].

Swept-source OCT (SSOCT) is an advanced imaging technique with better resolution and tissue penetration that can provide more detailed images of the deeper ocular structures like the choroid and even the sclera [53,54,55].

An interesting study by Filloy et al. compared SSOCT with traditional ultrasound imaging, highlighting the consistent accuracy of SSOCT in measuring choroidal tumor diameter and effectively distinguishing the lesion from surrounding normal choroid. The study also showcased the diagnostic utility of SSOCT in CCHs, revealing a unique spongelike pattern corresponding to vascular structures within the tumor. Utilizing SSOCT to examine DCHs could further deepen our comprehension of this condition [65,66,67].

**Figure 4:**
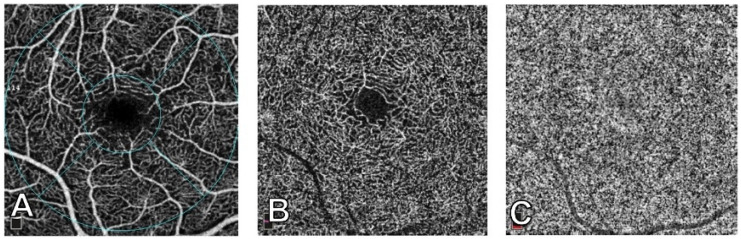
3 mm × 3 mm images of macular perfusion using optical coherence tomography angiography. (**A**) Superficial capillary plexus, showing the early treatment diabetic retinopathy study contour areas: Fovea (central circle with a 1 mm radius), temporal, superior, nasal, and inferior. (**B**) Deep capillary plexus. (**C**) Choriocapillaris. From [68].

Optical coherence tomography angiography (OCTA) is a novel technique that works on detecting motion, specifically focusing on vascular flow by monitoring erythrocyte movement. It captures multiple images, which are then analyzed by OCTA software to identify motion by comparing sequential scans. This technology enables the segmentation of choroid-retinal vascularization into distinct layers, including the superficial capillary plexus (SCP) and the deep capillary plexuses (DCP), the outer retina, and the choriocapillaris (CC). Figure 4 shows the OCTA segmentation into SCP, DCP, and CC layers [68,69].

A recent study examined the role of OCTA in evaluating CCHs, underlying how it can provide insight into lesion characteristics by analyzing density, flow, and vascular patterns. Differentiated vascular patterns, like worm-like, spaghetti-shaped, and club-shaped, have been identified within CCHs using OCTA [70,71]. OCTA serves as an indicator of the activity of lesions. Active lesions often display a dominant club-shaped vascular design in the deep choroidal layers, whereas areas with absent signals point to inactive lesions. Furthermore, in CCHs, OCTA is beneficial in monitoring the results of treatments and evaluating approaches such as plaque brachytherapy and laser photocoagulation [72,73].

It would be interesting to verify if these findings in CCHs are also applicable to DCH, therefore further studies are necessary to investigate the use of OCTA as a complement to other imaging methods like FFA and ICGA in the diagnosis of a DCH in SWS.

### 2.7. Near-Infrared Reflectance (NIR)

Near-infrared reflectance (NIR) imaging studies the photoreceptors, the RPE, and the choroid. It functions using a diode laser with an extended excitation wavelength of approximately 820 nm. This extended wavelength facilitates enhanced penetration through the optical media of the eye, enabling a comprehensive study of the retina and choroid [74]. Notably, while NIR imaging has been applied with promising results in diverse contexts, its utility for evaluating DCHs has been less explored. In a case study involving an 8-year-old patient, NIR imaging unveiled a DCH displaying numerous hyperreflective dots inside hyporeflective circles. These modifications concurred with the appearance of white dot-shaped “micro drusen-like” changes observed on SDOCT cross-sectional images. This showcases the ability of NIR imaging to unveil subtle intricacies within DCH, introducing novel insights [40,74]. The growing potential of NIR imaging in delineating DCHs underscores the need for further dedicated studies, particularly in the context of pediatric patients where non-invasive and rapid imaging techniques like NIR play a pivotal role in monitoring conditions over time [40,74].

### 2.8. Ultrasound Imaging

**Figure 5:**
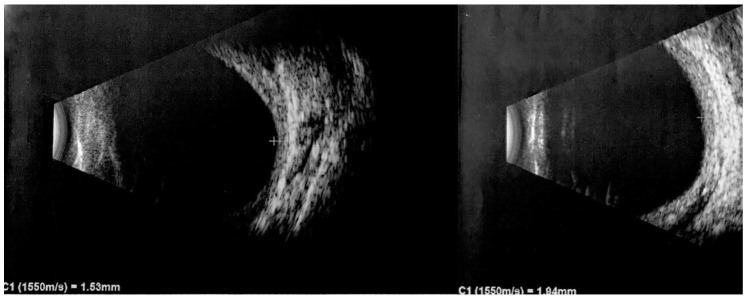
B-scan ultrasound examination. Diffuse choroidal hemangioma in both eyes, especially in the left eye (LE). Choroidal thickness of 1.53 mm and 1.94 mm in the right eye (RE) and LE, respectively. From [6].

Ultrasound is a nonintrusive imaging method for diagnosing and monitoring DCHs in SWS. Ocular ultrasonography employs high-frequency ultrasound waves to produce detailed images of the eye and adjacent structures. A-Scan ultrasonography uses a single ultrasound beam, providing one-dimensional information predominantly used for biometric evaluations, including intraocular lens calculations, and assessing the axial length. It typically operates in the 7–10 MHz frequency range. In contrast, B-Scan ultrasonography, usually working between 10 and 20 MHz, uses multiple ultrasound beams to generate a two-dimensional cross-sectional view of the internal ocular structures, crucial for examining the posterior segment when visual inspection is impeded [75]. Upon US imaging, DCHs reveal a thickened choroid with pronounced internal reflectivity, as illustrated in Figure 5. This important feature differentiates it from choroidal melanoma, which typically exhibits low internal reflectivity [76,77].

Additionally, DCHs demonstrate a regular internal structure and lack significant vascularization [65]. In B-mode, the ultrasound image portrays a solid, dome-shaped lesion with hyperechogenicity and lacks a posterior shadow. This imaging technique is also important for confirming the presence of associated features like retinal detachment and superficial calcifications [13]. However, ultrasound also has certain limitations. It provides two-dimensional images and may not capture the full extent of complex hemangiomas or small lesions. In such cases, additional imaging modalities like FFA and ICGA may be necessary to complement the ultrasound findings.

Ultrasound imaging techniques offer a comprehensive and reliable means to identify and differentiate DCH, contributing to effective clinical management and treatment planning. The procedure is cost-effective and relatively non-invasive. It is less invasive than FFA and ICGA, which require intravenous dyes; however, it is more operator-dependent and fastidious to the young patient than faster-acquiring techniques such as SDOCT. An overview of the main characteristics of each imaging modality applicable for the diagnosis of a DCH can be found in Table 1.

### 2.9. Treatment Strategies for DCHs

Asymptomatic DCHs without subretinal fluid should be monitored over time and generally does not require treatment. SDOCT imaging is particularly important in detecting even the smallest collection of subretinal fluid and prompting ICGA examination to plan treatment strategies such as laser photocoagulation of leaking vessels or verteporfin photodynamic therapy (PDT) [43,82].

PDT is a preferred option in CCHs and, more recently, has emerged as a promising treatment for DCH, primarily if associated with extensive retinal detachment. PDT selectively destroys abnormal vessels while preserving retinal structures. However, outcomes vary, with retinal detachment resolution seen in some cases and subretinal fluid persistence in others. Adjunct therapies like vascular endothelial growth factor (VEGF) inhibitors, such as bevacizumab, have also been explored to potentially reverse VEGF level increases following PDT treatment [43,82,83].

Further therapeutic options are proton beam therapy, targeted radiation using ruthenium106, cobalt60 plaque brachytherapy, transpupillary thermotherapy, and gamma knife radiosurgery. These strategies are particularly useful when DCHs are enlarging and present a persistent subretinal fluid, whereas extensive retinal detachment may require scleral buckling surgery with vitrectomy and gas injection. Oral propranolol efficacy varies in the literature [45,84,85,86,87,88].

Close monitoring and collaboration between medical retina specialists, surgical retina specialists, and ocular oncologists who are familiar with therapies such as proton beam therapy and brachytherapy are crucial in achieving optimal outcomes in managing DCH complications in SWS patients.

## 3. Conclusions

DCHs in SWS demand a comprehensive approach that leverages diverse imaging modalities. This multimodal strategy provides crucial insight into DCH diagnosis, progression, and management. Visiting patients with SWS can be somewhat challenging due to the young age of patients at the time of diagnosis, possible cognitive impairment, possible anterior segment opacities related to previous glaucoma surgeries, and the subtle appearance of a DCH on fundus examination. Therefore, a multimodal imaging approach can help ophthalmologists in comprehensively evaluating this pathology.

Fundus photography facilitates rapid documentation of characteristic features such as color variation. NIR imaging uncovers subtle RPE variations. FFA and ICGA offer distinct views of retinal vascular dynamics and the choroidal circulation. SDOCT has revolutionized imaging by offering high-resolution cross-sectional views of retinal and choroidal layers and EDI technology allows better choroidal thickness measurement and the detection of additional DCH features. Ultrasound imaging completes the multimodal approach with A-scan and B-scan modes that provide insights into tissue characteristics and ocular structures, distinguishing DCHs from other conditions. This integrative imaging approach not only aids in diagnosis but also guides treatment planning and disease management for affected individuals. Fundus photography, FA, NIR imaging, SDOCT, and ultrasound imaging are non-invasive techniques, which can be repeated during successive follow-ups and are important in excluding retinal complications such as subretinal fluid or retinal detachment that can potentially arise over time. FFA and ICGA are more invasive, as they require the administration of intravenous dye, and thus can be more important in cases in which other imaging modalities detect an enlargement of the DCH, or in patients who need to undergo laser photocoagulation or PDT to identify vessel leakage and monitor post-operative vessel atrophy.

Recent advancements in ophthalmic imaging technologies, such as OCTA, offer a non-invasive and comprehensive approach to assessing the characteristics of DCH. The capability of OCTA to evaluate vascular patterns introduces exciting prospects for further research. The ability of current optical coherence tomography technology, which enables the visualization of critical interfaces including the challenging choroidal-scleral boundary, is a valuable tool for investigating DCH.

Future prospective research could focus on the enhancement of SDOCT technology, which is constantly in evolution, and the integration of automated detection of the choroidal-scleral junction and automatic measurement of the choroidal thickness to facilitate the detection of subtle changes. Furthermore, in addition to assessing choroidal thickness, the exploration of the choroidal vascularity index (CVI) could hold promise for offering more comprehensive insights into DCH. CVI, which indicates the ratio of the luminal area to the total choroidal area, provides a deeper understanding of choroidal vascularity. The integration of these advanced technologies holds the potential to improve our understanding and management of DCHs in SWS [89]. Multimodal imaging techniques empower clinicians to accurately diagnose DCH, assess its progression, and tailor treatment strategies. While each method offers unique insights, their combined use enhances diagnostic accuracy and guides therapeutic interventions.

## Figures and Tables

**Table 1:** Summary of advantages, disadvantages, technical characteristics, and appearance of DCHs with different imaging modalities.

Imaging Modality Technical Characteristics	Advantages	Disadvantages	DCH Appearance
Fundus Photography WL 490–615 nm [78] FOV 45°–200° [78]	Non-invasive; ease of capturing images; both eyes can be assessed simultaneously.	The extent or thickness of the hemangioma is not assessable. Shallow DCH does not significantly alter the fundus appearance.	DCH’s subtle color differences known as “tomato ketchup” appearance. Additional characteristics such as exudative retinal detachments, venous congestion, or hyperplastic RPE changes can be monitored.
FA WL 488–514 nm [79] FOV 30°–200° [80]	Efficient; non-invasive; useful in evaluating the presence of subretinal fluid or retinal detachment.	The extent or thickness of the hemangioma is not assessable.	Untreated DCH exhibits intrinsic iso-FA or hypo-FA patterns; treated DCH often displays a higher prevalence of hypo-FA traits. Extrinsic DCH findings including RPE hyperplasia, atrophy, and fibrous metaplasia.
NIR WL 790 nm [79] FOV 30–200° [80]	Efficient; non-invasive.	Further studies for its implication in DCH’s diagnosis and monitoring are necessary.	Greater tissue penetration of NIR allows rapid evaluation of the EPR. A rare case of hyperreflective dots was reported.
FFA WL 465–530 nm [81] FOV 55°–200° [81]	FFA can be particularly informative in cases where DCH is associated with retinal detachment.	It requires dye administration which rarely can give rise to allergic reactions. It can interfere with renal and hepatic metabolisms. Requires substantial patient collaboration.	FFA has been shown to depict early hyperfluorescence and subsequent late-phase leakage consistent with exudative retinal detachment linked to DCH.
ICGA WL 790–835 nm [57] FOV 55°–105° [81]	ICGA offers a detailed dynamic visualization of DCH; valuable insights into the intricate choroidal circulation within DCH.	Requires dye administration, which rarely can give rise to allergic reactions. It can interfere with renal and hepatic metabolisms. Requires substantial patient collaboration.	Early phase: vascular network within the hemangioma is quickly filled. Intermediate phase: complete dye filling yields intense hyperfluorescence with a mix of hyper- and hypofluorescent dots on an already luminescent background. Late phase: pronounced hypofluorescence of the tumor when compared to the adjacent choroid emerges as a signature feature.
SDOCT WL 840–870 nm [56,57,58,59].	Efficient; non-invasive; allows for the identification of retinal layers and assessment of retinal thickness.	In cases of thickened choroid SDOCT falls short in evaluating the choroidal-scleral junction.	It can reveal the breakdown of Bruch’s membrane and RPE, intraretinal fluid, and subretinal fluid.
EDI-SDOCT WL 840–870 nm [56,57,58,59].	Efficient; non-invasive; allows for a better visualization of the choroidal-scleral junction which renders manual choroidal thickness measurement easier.	Not always available in hospital settings.	Choroidal vasculature is visualized on EDI-SDOCT cross sectional images. Profile alterations of the retina and choroid are well visible.
SSOCT WL 1055–1300 nm [65,66,67].	Efficient; non-invasive; allows for a better visualization of the choroidal-scleral junction which renders manual choroidal thickness measurement easier.	Expensive technology, rarely available in hospital settings.	Consistent accuracy of SSOCT in measuring hemangioma thickness and effectively distinguishing the lesion from surrounding normal choroid. Profile alterations of the retina and choroid are better evaluated.
OCTA WL 840–870 nm [56,57,58,59].	Efficient; non-invasive; it provides insights into lesion characteristics by analyzing density, flow, and vascular patterns.	Further studies for its implication in DCH diagnosis and monitoring are necessary.	Differentiated vascular patterns, like worm-like, spaghetti-shaped, and club-shaped, have been identified within CCH using OCTA.
US Frequency: A-scan 7–10 MHz B-scan 10–20 MHz [75]	Cost effective, efficient. Helps in distinguishing DCH from malignant choroidal tumors such as melanomas.	It provides two-dimensional images and may not capture the full extent of complex hemangiomas or small lesions.	Benign tumors reveal a thickened choroid with pronounced internal reflectivity. DCH demonstrates a regular internal structure and lacks significant vascularization. DCH appears as a solid, dome-shaped lesion with hyperechogenicity and lacks a posterior shadow. Detects presence of associated features like retinal detachment and superficial calcifications.

DCH: diffuse choroidal hemangioma; WL: wavelength; FOV: field of view; RPE: retinal pigment epithelium; FA: fundus autofluorescence; NIR: near-infrared reflectance; FFA: fluorescein angiography; ICGA: indocianine green angiography; SDOCT: spectral domain optic coherence tomography; EDI-SDOCT: enhanced depth imaging-SDOCT; SSOCT: swept-source OCT; OCTA: optic coherence tomography angiography; US: ultrasound.

## Data Availability

Not applicable.
